# Physical nature of intermolecular interactions inside Sir2 homolog active site: molecular dynamics and ab initio study

**DOI:** 10.1007/s00894-016-2992-2

**Published:** 2016-05-06

**Authors:** Przemysław Czeleń, Żaneta Czyżnikowska

**Affiliations:** Department of Physical Chemistry, Collegium Medicum, Nicolaus Copernicus University, Kurpińskiego 5, 85-950 Bydgoszcz, Poland; Department of Inorganic Chemistry, Wrocław Medical University, Borowska 211, 55-556 Wrocław, Poland

**Keywords:** Docking procedure, Molecular dynamic simulations, Intermolecular interaction energy partitioning

## Abstract

In the present study, we analyze the interactions of NAD+-dependent deacetylase (Sir2 homolog yeast Hst2) with carba-nicotinamide-adenine-dinucleotide (ADP-HPD). For the Sir2 homolog, a yeast Hst2 docking procedure was applied. The structure of the protein–ADP-HPD complex obtained during the docking procedure was used as a starting point for molecular dynamics simulation. The intermolecular interaction energy partitioning was performed for protein–ADP-HPD complex resulting from molecular dynamics simulation. The analysis was performed for ADP-HPD and 15 amino acids forming a deacetylase binding pocket. Although the results indicate that the first-order electrostatic interaction energy is substantial, the presence of multiple hydrogen bonds in investigated complexes can lead to significant value of induction component.

## Introduction

Weak noncovalent interactions involving molecules of biological importance have been identified to play an important role in many fundamental processes in nature. The detailed analysis of strength of hydrogen bonds and London dispersion interactions can provide a better understanding of the structure and stability of biomolecules [1]. The forces are responsible for specificity of DNA-protein binding. Many pharmaceutical ligand–protein interactions are noncovalent in their nature. Although the X-ray measurements results are often available and give a lot of geometrical information, the magnitude and character of interaction between the substrate-specific ligand and enzymes need further exploration.

**Fig. 1:**
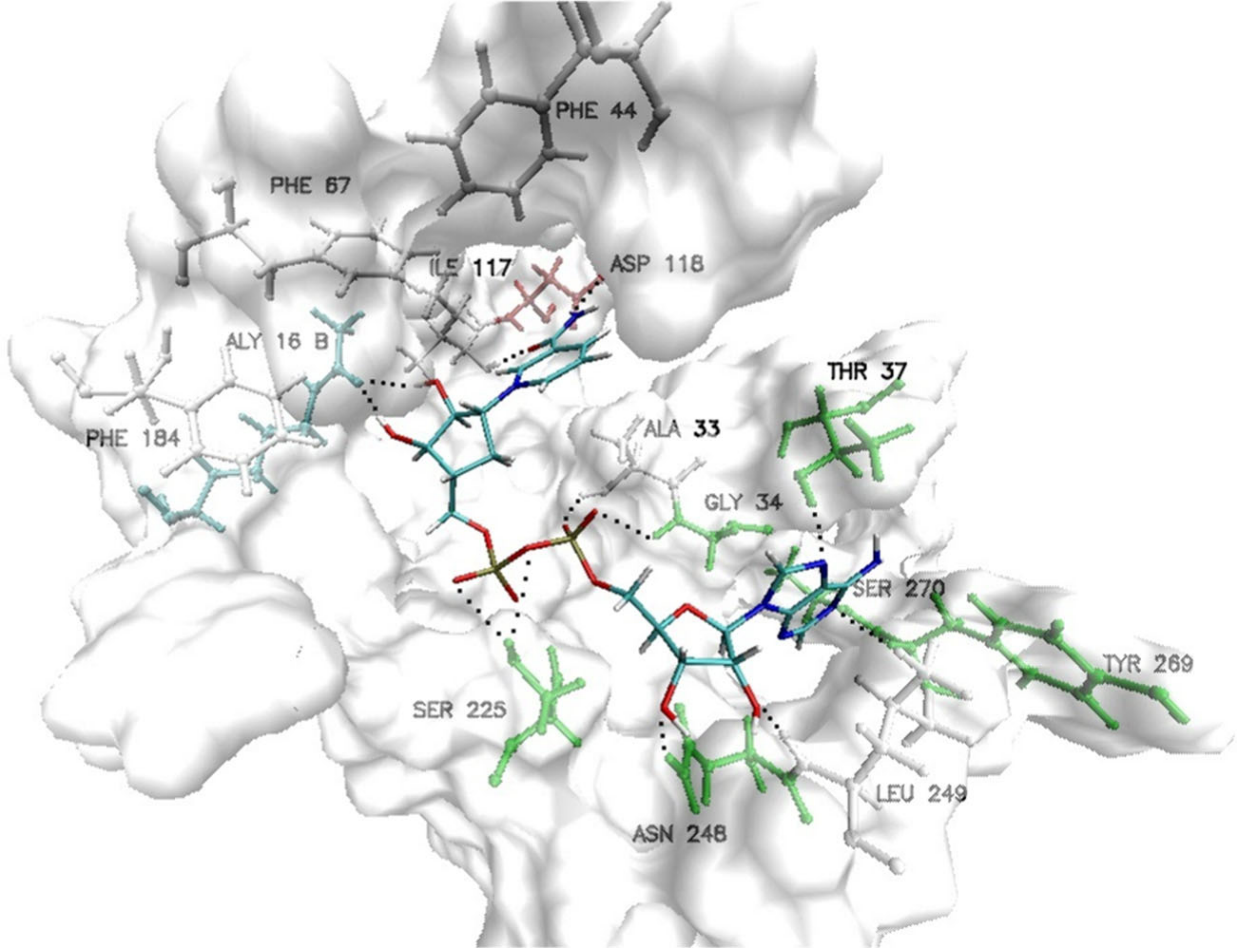
Graphic representation of Hst2 active site with ADP-HPD. Marked amino acids play the most important role in creation of interactions with ligands

Due to their biochemical properties Sir2 (silent information regulator 2) proteins are involved in various biological processes such as DNA metabolism, regulation, and repair of double-stranded breaks [2, 3]. This group of proteins can be involved in transcriptional silencing, apoptosis and chromosome stability. Their promoting activity in longevity in yeast mother cells was also proved [4]. It has been also indicated that some Sir2 homologs in eukaryotes have been implicated in the proper cell cycle progression, radiation resistance, and genomic stability [5]. To date, investigations strongly suggest that their biological activity is largely dependent on their deacetylate properties and is also partially modulated by concentration of nicotinamide in cells. The conformation of Sir2 is unique among all Sir proteins and was carefully determined [6]. In the current study we present the structural and energetical consequences of interactions between NAD+-dependent deacetylase (Sir2) with carba-nicotinamide-adenine-dinucleotide (ADP-HPD) [7]. According to Sanders’ study, nicotinamide molecule is bound to the D-pocket which has hydrophobic character due to the PHE67, PHE184, around the pyridine ring and PHE44 together with ILE117 proximal to the carboxamide moiety (See Fig. 1). The docking procedure and molecular dynamics simulations were performed in order to gain an insight into the structural and energetical basis of Sir2 enzyme inhibition. Moreover, the total intermolecular energy between protein and ADP-HPD was divided into physically relevant terms determining the nature of interactions in complex in question.

## Methods

### Docking procedure

**Fig. 2:**
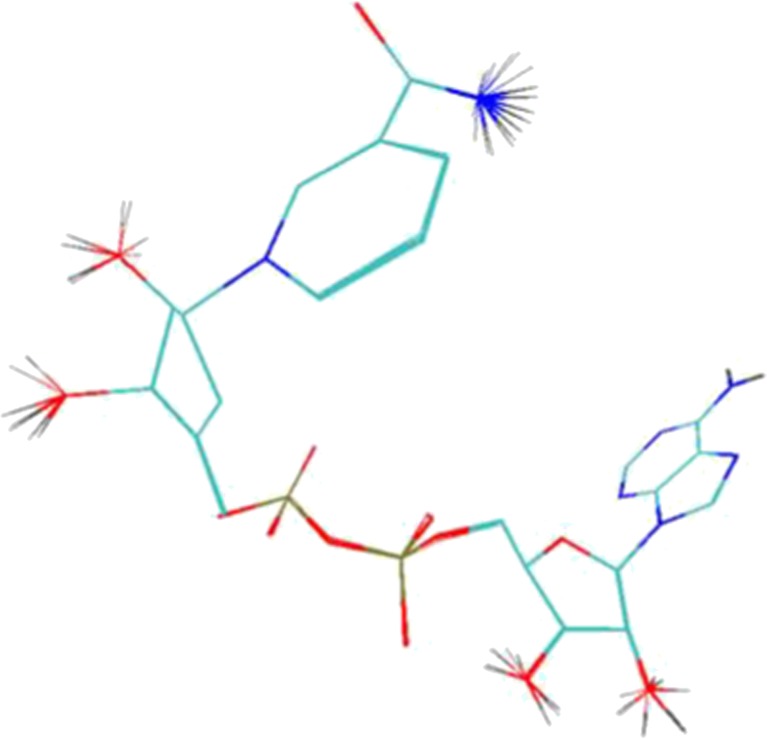
ADP-HPD ligand conformations obtained during docking procedure

During the docking procedure, the structure of the Sir2 homolog with an acetyllysine histone H4 peptide yeast Hst2 was used. The structural data of the considered protein, denoted as 1SZC [8], was downloaded from the Protein Data Bank. Both structures used during the docking procedure, namely Sir2 protein and optimized ADP-HPD ligand, contained only polar hydrogen atoms. The docking stage was performed with use of the united-atom scoring function implemented in AutoDock Vina [9]. The space declared during the docking procedure (18 × 20 × 24) has been limited to the active site of Sir2 enzyme. The docking procedure for considered subunits was repeated ten times with exhaustiveness equal 30; its outcomes are presented in Fig. 2. In all cases, atoms of ADP-HPD ligand backbone are localized almost in this same point of conformational space of the active site. Small differences are mainly related to localization of hydrogen atoms.

### Molecular dynamics simulation

**Fig. 3:**
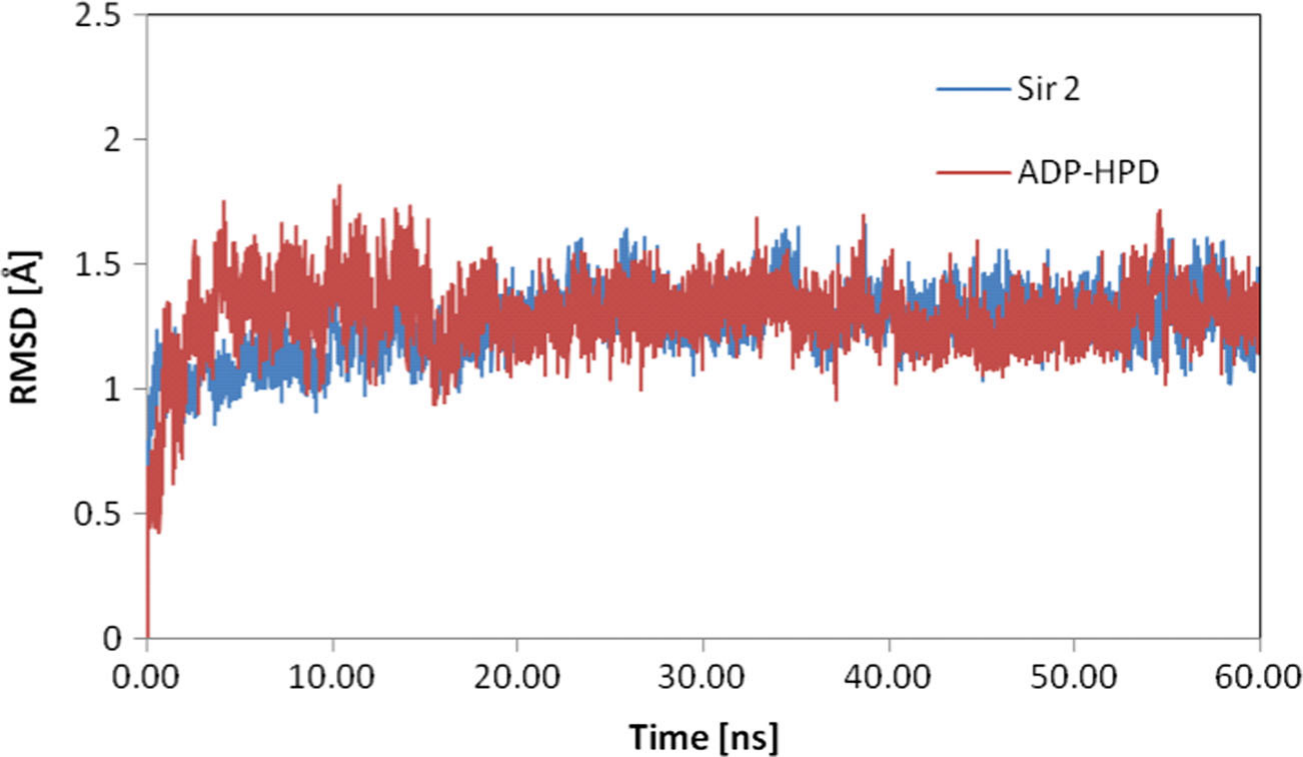
Distribution of RMSD values for Sir 2 protein and ADP-HPD ligand during molecular dynamics simulation

The structure of the Hst2–ADP-HPD complex obtained during docking procedure was used as a starting point for molecular dynamics simulation. Ligand structure was characterized with the use of amber force field parameters and the atomic charges were calculated according to the Merz–Kollman scheme via the RESP procedure [10] at HF/6-31G* level. The complex structure was neutralized and immersed in a periodic box of a TIP3P water box [11]. The analyzed system was heated to 300 K during 100 ps of MD simulation and the temperature was controlled by a Langevin thermostat [12]. The periodic boundary conditions and SHAKE algorithm [13] were applied for 60 ns of molecular dynamic simulation. The analysis of molecular dynamics simulation parameters, namely potential and kinetic energies of system and RMSD values, confirm that the first 20 ns of obtained trajectory is an equilibration period. Values presented in Fig. 3 confirm that both elements of the considered system, namely protein and ADP-HPD ligand, after this stage reached dynamic stability. The final 40 ns of trajectory, collected during molecular dynamics simulation, was used in analysis of the interaction between considered subunits. Structural analysis was performed with use of the VMD package [14]. In all molecular dynamics simulations, the AMBER 11 package was used [15].

### Intermolecular interaction energy partitioning

Intermolecular interaction energy partitioning was performed for the protein–ADP-HPD complex resulting from molecular dynamics simulation. The analysis was performed for ADP-HPD and amino acids forming a deacetylase binding pocket. In the present study, we discuss the results obtained using the variational-perturbational scheme implemented in a modified version of the GAMESS US package [16, 17].

In this approach, the total intermolecular interaction energy calculated at Hartree–Fock level of theory (*Δ*E^HF^) is decomposed into the first–order electrostatic (*ε*_el_^(10)^), Heitler–London exchange (*Δ*E_ex_^HL^) and delocalization components (*Δ*E_del_^HF^). The delocalization term, also referred to as the deformation component, contains charge-transfer and polarization effects. Since the dimer-centered basis set is employed during the calculations, both the total intermolecular interaction energy as well as its components are free from the basis set superposition error.

$$ \varDelta {E}^{HF}={\varepsilon}_{el}^{\left[10\right]}+\varDelta {E}_{ex}^{HL}+\varDelta {E}_{del}^{HF} $$

The analysis was performed for ADP-HPD and 15 amino acids forming a deacetylase binding pocket. The RHF/aug-cc-pVDZ level of theory and about 1700 contracted Cartesian Gaussian basis functions were employed to describe the systems. Due to the size of the studied complexes, we have not attempted to determine interaction energy components resulting from the partition of the correlation energy at the MP2 level of theory (i.e., uncoupled dispersion energy, correlation correction to electrostatic energy and exchange–delocalization term) [18].

## Results and discussion

### Docking and molecular dynamics simulations

**Fig. 4:**
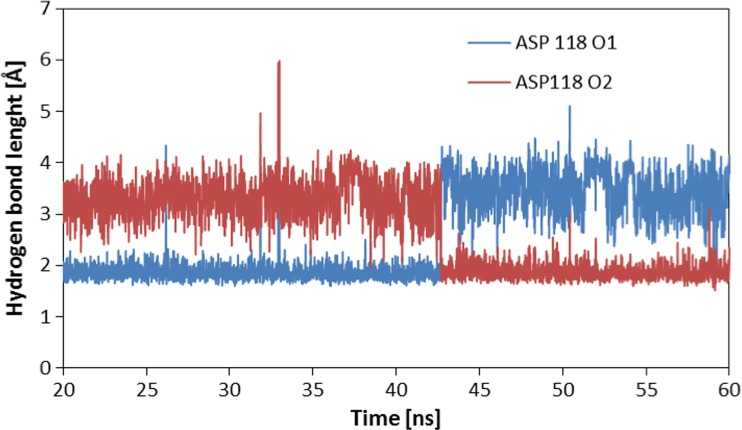
Distribution of hydrogen bonds length created by oxygen atoms of ASP118 with ADP-HPD ligand

The docking procedure allows for a precise definition of structure of analyzed complex and interactions between both subunits. For all conformations obtained during the docking stage, the atoms of ADP-HPD ligand backbone are localized almost in this same points of conformational space of active site. Small differences are mainly related to localization of hydrogen atoms. The average value of binding affinity of the ligand obtained during this stage is −12.15 kcal/mol and maximum differences between considered conformations do not exceed 0.1 kcal/mol. An orientation of ADP-HPD in the enzyme active site determined at this stage exhibits a high similarity relative to experimental structure. Stability of this complex is maintained by numerous interactions among which hydrogen bonds play an important role. The carba-nicotinamide part of the ligand is stabilized by interactions created with ILE117, ASP118, and ALY16 B, adenine part of considered molecule creates bonds with ASN248, LEU249, THR37 and SER270, also oxygens from orthophosphoric part creates interactions with ALA33 and SER225. The trajectory obtained during 40 ns of equilibrated molecular dynamic simulation allowed evaluating the stability of the considered interactions. The values listed in Table 1 present distribution of hydrogen bond length for all conformations collected during the simulation. Bonds created by ADP-HPD with ILE117 and ASP118 are stable over all simulation times, in the case of bonds created by first amino acid interactions of medium and weak binding power dominate, while ASP118 creates mainly strong hydrogen bonds. Both oxygen atoms from rotatable carboxyl group play important roles in creation of interactions with ADP-HPD. The values presented in Fig. 4 unambiguously confirm that during whole simulation always one of oxygens atoms, from carboxyl group of aspartic acid, creates stable and strong hydrogen bond with hydrogen atom from nicotinamide part of ligand. Also, the adenine part of considered ligand creates stable hydrogen bonds. The most important interactions involve ASN248 and LEU249. In the case of first of mentioned amino acids, hydrogen bonds are present by 90 % of simulation time, most of them is characterized by strong and medium binding power. Bindings created by LEU249 are observed in 95 % of trajectory steps, their lengths indicate the dominant contribution of medium and weak interactions. The next non-negligible hydrogen bonds are created by SER270 and THR37 but in both cases frequency of occurring and binding power is much smaller than in the case of ASN248 and LEU249. The middle orthophosphoric part of ADP-HPD is stabilized by interactions, of strong and middle binding power, involving ALA33. Presented distribution of hydrogen bonds indicate on small mobility of ADP-HPD in conformational space of active site what is confirmed also by stable RMSD values (av. 1.23 ± 0.18).

**Table 1:** Frequencies of hydrogen bond occurrence between ADP-HPD and amino acids from active site during molecular dynamics simulations

	Amino acids	Hydrogen bond length [Å]							
		1.625	1.875	2.125	2.375	2.625	2.875	3.125	3.375
Frequency of occurrence [%]	ILE 117	0.5	15.0	32.7	27.2	14.4	7.2	2.1	0.7
	ASP 118(O1)	9.1	35.2	6.3	0.6	1.3	3.0	5.5	7.8
	ASP 118(O2)	9.0	32.8	5.7	1.2	2.8	8.1	12.1	12.7
	ALY 16 B	13.6	19.6	6.2	1.7	1.7	1.5	1.3	1.4
	ASN 248	0.4	24.8	37.5	17.3	6.2	3.0	2.0	1.5
	SER 270	0.2	9.9	18.6	16.8	17.2	18.2	12.0	3.9
	LEU 249	0.1	11.8	34.2	26.6	13.4	7.5	2.8	1.7
	THR37	1.0	17.3	16.0	6.1	2.7	3.1	3.4	3.4
	ALA 33	0.8	25.7	34.4	15.2	5.2	1.5	0.5	0.4
	SER 225	0.0	0.0	0.0	0.7	5.2	17.1	26.5	22.7

Values of hydrogen bond length represent middle values of intervals with a width of 0.25 Å

**Fig. 5:**
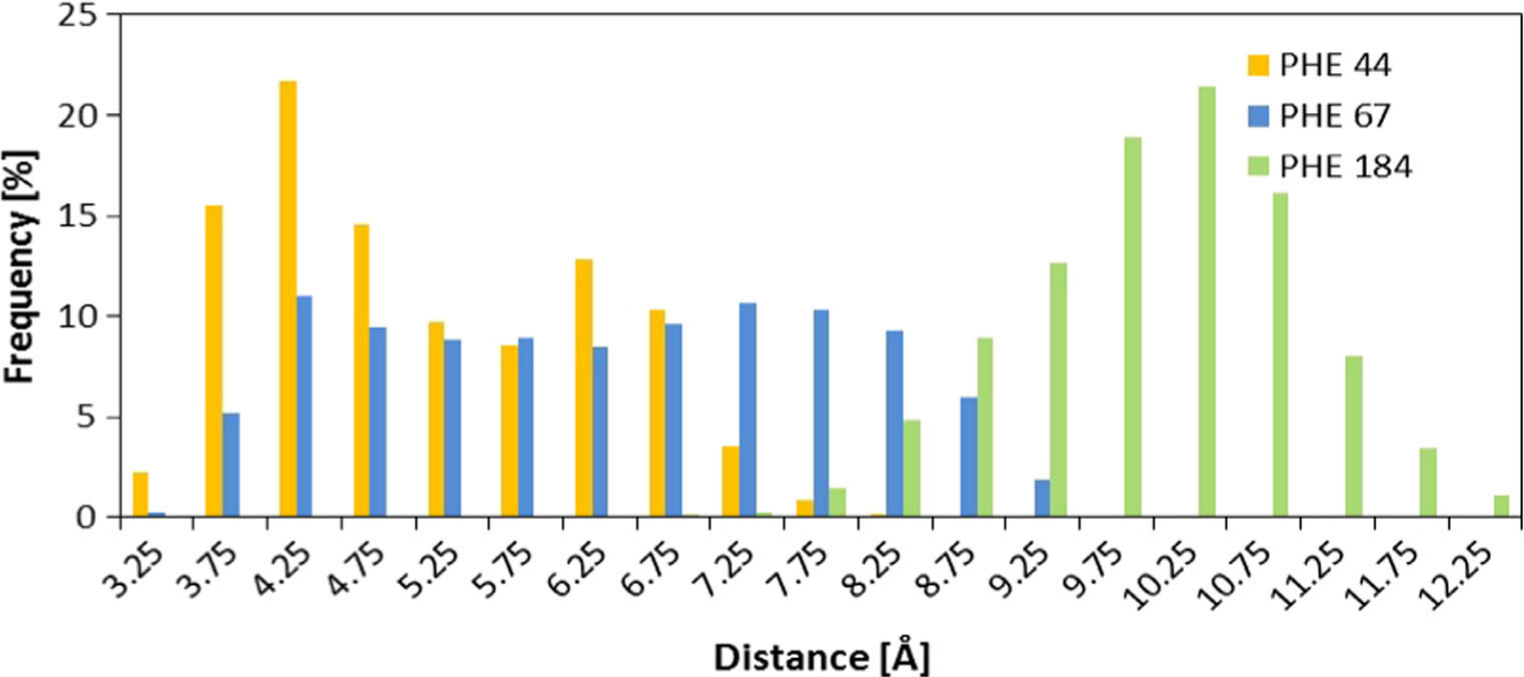
Distribution of distances between carbon atoms of phenylalanines and nicotinamide part of ADP-HPD ligand

The next group of important forces related to stabilization of considered ligand is hydrophobic interactions. Active site of Sir 2 contains many amino acids, which could be involved in such interactions. The most important role can be fulfilled by aromatic amino acids localized in the vicinity of heterocyclic rings of ADP-HPD, namely phenyloalanies PHE44, PHE67, and PHE184. Figure 5 presents distances between aromatic systems of amino acids and nicotinamide part of considered ligand during molecular dynamics simulations. Presented distributions indicate that at least one of considered phenylalanine moieties plays an important role in stabilization of the ADP-HPD molecule. In the case of PHE44 by 50 % of simulation time, the distance between aromatic rings does not exceed 5 Å, and mutual orientations between considered molecules allow the occurrence of stacking interactions. Also, localization of PHE67 in the active site during simulation ensures a large group of conformations, enabling the occurrence of hydrophobic interactions with heterocyclic ring of ADP-HPD.

### Selection criteria

During this stage we selected the structure of the complex of ADP-HPD with Sir2 protein, which should be the most representative relative to the conformations occurring during considered simulation times. For this purpose, we used structural data describing distributions of 11 of the most important hydrogen bonds and distances between aromatic rings of the considered subunits. Several criteria were considered to choose a representative structure. One of the conditions that should fulfill the chosen structure was the smallest total value of considered hydrogen bonds, which should potentially correlate with the highest affinity of ligand toward active site. The second criteria required that hydrogen bond length and also distance between the considered aromatic subunits should belong to the most-represented intervals of distributions obtained during simulation times.

### Intermolecular interaction energy partitioning

The existence of multiple hydrogen bonds between the ADP-HPD molecule and the surrounding environment suggests that electrostatics should be important. Indeed, in terms of absolute numbers, its value is quite large, and has mainly a stabilizing character. Its highest value was found for the positively charged ARG45-ADP-HDP complex. As might be expected, the lowest values of electrostatic component (close to 0 kcal/mol) were observed for complexes formed by the aromatic ring of phenylalanine and ADP-HDP molecule. As is shown in Table 2, in the case of some complexes, the stabilization due to the mutual polarization of interacting subsystems reflected in the negative value of delocalization component (encompassing induction terms) and is also quite important. In the case of complexes containing ALA33 and ASP118, stabilization originates strongly from the interaction of permanent and induced multipole moments of subsystems due to their mutual polarization. In the case of phenylalanine and arginine complexes, the effects resulting from quantum-mechanical exchange of electrons between subsystems are particularly small. Some results indicate that although the first–order electrostatic is substantial, it might be completely quenched by associated Pauli repulsion. It was observed, for example, for weak interactions of ASN245 and TYR269 with ADP-HDP molecules. Consequently, it would be interesting to estimate the correlation contributions to the total intermolecular stabilization.

**Table 2:** Intermolecular interaction energy partitioning

ADP-HPD-amino acid	Intermolecular interaction energy components				
	DE^HL^	e_el_^(10)^	DE_ex_^HL^	DE_del_^HF^	DE^HF^
ARG 45	−28.74	−29.07	0.33	−3.09	−31.83
ALA 33	−5.90	−18.02	12.12	−10.51	−16.41
ASN 248	2.22	−8.18	10.40	−3.76	−1.54
GLY 34	0.08	−5.09	5.17	−2.63	−2.55
LEU 249	2.1	−12.4	14.47	−3.3	−1,2
PHE 184	−0.80	−1.20	0.39	−0.66	−1.46
SER 270	3.74	−9.408	13.15	−4.34	−0.60
PHE 44	1.04	−0.10	1.14	−0.29	0.74
PHE 67	0.66	−0.44	1.10	−0.22	0.44
ALY 16 B	−4.15	−15.64	11.48	−5.15	−9.30
ILE 117	2.39	−5.86	8.26	−2.08	0.32
THR 37	1.97	−16.81	18.79	−6.95	−4.94
SER 225	3.68	−8.24	11.92	−7.47	−3.80
ASP 118	13.59	−9.85	23.44	−12.68	0.90
TYR 269	0.71	−3.74	4.43	−1.37	−0.658
ADP	−17.78	−107.18	89.40	−34.85	−52.62
NICO	14.62	−14.54	29.16	−12.72	1.90

The data are given in kcal/mol

In order to characterize the relationships between intermolecular interaction energy components, Spearman rank correlation was used [19].

$$ \rho =1-6{\displaystyle \sum {d}_i^2/n\left({n}^2-1\right)} $$

The data collected in Table 3 were obtained using the expression above, where **d** is the difference between ranks of the compared terms and **n** is the number of investigated complexes. It is seen that among of all pairs of components, the correlation coefficients of ΔEel/ΔEdel and ΔEel/ΔEHF are high. Between the exchange and delocalization components, an evident negative correlation with coefficient equal to −0.8 is observed (Table 4).

**Table 3:** Spearman rank correlation coefficients estimated for intermolecular interaction energy components for considered pair

	e_el_^(10)^	ΔE_ex_^HL^	ΔE_del_^HF^	ΔE^HF^
ΔE^HL^	0.14	0.6	−0.3	0.6
e_el_^(10)^		−0.5	**0.76**	**0.7**
ΔE_ex_^HL^			**−0.8**	0.025
ΔE_del_^HF^				0.4

**Table 4:** Properties of selected complexes

Complex	Distance	Interaction energy	DE_del_^HF^/e_el_^(10)^
ILE 117 N-H…O	2.03	0.32	0.35
ALY 16 B O…H-O	1.93	−9.30	0.33
ASN 248 N-H…O	2.06	−1.54	0.46
SER 270 O-H…N	2.13	−0.60	0.46
LEU 249 N-H…O	1.94	−1.2	0.27
THR 37 O-H…N	1.82	−4.94	0.41
ALA 33 N-H…O	2.01	−16.41	0.58
SER 225 N-H…O	2.07	−3.80	0.90

Distances (Å), intermolecular interaction energies (kcal/mol), DE_del_^HF^/e_el_^(10)^ ratio

The nature of the hydrogen bonds stabilizing of investigated system can be characterized based on the theory of intermolecular interactions. Since the higher-order delocalization effects involve charge transfer excitation, they can be associated with covalent character of interactions. It was also shown by Grabowski et al. that delocalization has a dominant stabilizing character in the group of complexes with short dihydrogen bonds (distances close to 1 Å) [20]. As they observed for the remaining hydrogen bonds, electrostatic energy should be the most important. Based on the mentioned study, we can conclude that the hydrogen bonds that are partially covalent have an Edel/Eel ratio higher than 0.45. In the case of our systems, consequently, the most covalent characters have ALA33 and SER225 complexes.

## Conclusions

Each fragment of ADP-HPD ligand, namely nicotinamide, adenine nucleotides, and orthophosphoric chains, play important roles in stabilization of a complex with enzyme by numerous interactions of varying nature. The presented values describing time evolution of ADP-HPD molecule in the complex with Sir2 protein correlate with outcomes of docking procedure. Most all of the important interactions identified on the docking stage are present during molecular dynamics simulations. The greatest accumulation of interactions is observed in the space of D-pocket due to the presence of numerous hydrogen bonds (ILE117, ASP118, ALY16 B) and hydrophobic interactions (PHE44, PHE67), also in other regions of active site are important bindings present by most simulation time. Quite small and stable RMSD values of ADP-HPD molecule, supported by high frequency of bindings creation during whole simulation, indicate small mobility and limited conformational flexibility of considered ligands in the space of enzyme active site.

As is seen, even at the uncorrelated level of theory, the picture of interactions is quite complicated. Although in the case of the studied complex typical stacking motifs are not present between ADP-HPD molecules and neighboring amino acids, it follows from previous studies that one might expect non-negligible additional stabilization resulting from dispersion component of interaction energy [21]. The analysis of correlations between the intermolecular interaction components revealed existing dependence between positive and negative for ΔEel/ΔEdel and ΔEex/ΔEdel, respectively.
